# “Surprise” and the Bayesian Brain: Implications for Psychotherapy Theory and Practice

**DOI:** 10.3389/fpsyg.2019.00592

**Published:** 2019-03-28

**Authors:** Jeremy Holmes, Tobias Nolte

**Affiliations:** ^1^ University College London, Anna Freud National Centre for Children and Families, London, United Kingdom; ^2^ Department of Psychology, College of Life and Environmental Sciences, University of Exeter, Exeter, United Kingdom

**Keywords:** Bayesian brain, psychoanalysis, active inference, psychotherapy, free energy principle, mentalization

## Abstract

The free energy principle (FEP) has gained widespread interest and growing acceptance as a new paradigm of brain function, but has had little impact on the theory and practice of psychotherapy. The aim of this paper is to redress this. Brains rely on Bayesian inference during which “bottom-up” sensations are matched with “top-down” predictions. Discrepancies result in “prediction error.” The brain abhors informational “surprise,” which is minimized by (1) action enhancing the statistical likelihood of sensory samples, (2) revising inferences in the light of experience, updating “priors” to reality-aligned “posteriors,” and (3) optimizing the complexity of our generative models of a capricious world. In all three, free energy is converted to bound energy. In psychopathology energy either remains unbound, as in trauma and inhibition of agency, or manifests restricted, anachronistic “top-down” narratives. Psychotherapy fosters client agency, linguistic and practical. Temporary uncoupling bottom-up from top-down automatism and fostering scrutinized simulations sets a number of salutary processes in train. *Mentalising* enriches Bayesian inference, enabling experience and feeling states to be “metabolized” and assimilated. “Free association” enhances more inclusive sensory sampling, while dream analysis foregrounds salient emotional themes as “attractors.” FEP parallels with psychoanalytic theory are outlined, including Freud’s unpublished project, Bion’s “contact barrier” concept, the Fonagy/Target model of sexuality, Laplanche’s therapist as “enigmatic signifier,” and the role of projective identification. The therapy stimulates patients to become aware of and revise the priors’ they bring to interpersonal experience. In the therapeutic “duet for one,” the energy binding skills and non-partisan stance of the analyst help sufferers face trauma without being overwhelmed by psychic entropy. Overall, the FEP provides a sound theoretical basis for psychotherapy practice, training, and research.

## Introduction

It has been established beyond doubt that psychodynamic psychotherapy “works” (Leichsenring, 2008; Shedler, 2010; Leichsenring et al., 2015; Taylor, 2015). But how? Building on recent advances in computational neuroscience, the aim of this paper is to offer a heuristic that can help elucidate the underlying mechanisms by which psychotherapies alleviate psychological distress and illness.^1^

Schröedinger (1944) coined the term “negentropy” to characterize the complexity of living matter, i.e., its structured heterogeneity and order, in contrast to the entropy of the inanimate world under the sway of the second law of thermodynamics. Our approach is based on the “free energy” (FE) principle developed by Friston as one formulation of “the Bayesian Brain” (Friston, 2010; Hobson and Friston, 2012; Hohwy, 2013; Friston and Frith, 2015; Hopkins, 2016). Friston presupposes that the brain’s aim, like that of the organism as a whole, is to maintain homeostasis^2^ and resist the entropic forces of chaos and homogenization. To do this we–along with our fellow living creatures–need *information* about the environment, our place within it, and the likely outcomes of our actions. The past shapes our futures: based on prior experience, we make “top down” predictions about our sensory and interoceptive input, based on a model of how they were created.^3^ The discrepancy between these top-down predictions and the actuality–and accuracy–of bottom-up sensations is “prediction error.” *Via* perception and action, these unavoidable “errors” are “minimized” by converting *prior* beliefs into *posteriors*^4^ *(i.e., the newly assigned probability after the relevant evidence, the observed data, is taken into account)*. This process of Bayesian inference simulates past experience and ensures posterior beliefs align with newly sampled data.

Prediction error is inescapable for two reasons: first, we live in a constantly changing environment, and second, our sampling of that environment is subject to inaccuracy and misperception. But this “error” is all to the good –it is the very stuff that drives a continuous process of belief-updating and helps build adaptive models of the worlds (and bodies) we inhabit.

The free energy principle (FEP) regards creatures (like us) as self-organizing systems that resist a tendency to dissipation and disorder. This applies as much to the brain in its search for meaning (i.e., informational order) as it does to the body as a whole in its pursuit of physical structure and regulation (Friston, 2013). This informational slant on “entropy” equates to “surprise.”^5^ If an event is probable to a high degree, the surprise when it occurs is minimal and thus little new information is gained. We can therefore be regarded as creatures that place an upper bound on free energy by minimizing their surprise, or maximizing the evidence for their models of the world. This is sometimes known as self-evidencing (Hohwy, 2016). Free energy can be decomposed into *complexity* minus *accuracy*. Accuracy refers to our ability to predict sensations, while complexity reflects the degrees of freedom used to provide an accurate prediction. Model evidence increases by minimizing free energy. The accuracy of predictions rises, with a “concomitant increase in complexity so that increased model complexity is always licensed by an ability to make more accurate predictions” (Solms, 2019).

This *predictive coding visualises* the brain as engaged in neuronal–and, as we shall argue, conceptual–dynamics, that minimize free energy by working to reduce prediction errors. The latter are the difference between sensory input and predictions of that input based upon expectations about states of the world created by a pre-existing “generative model.” Resolving prediction errors updates prior beliefs by converting them into posterior beliefs. The empirical evidence from neuroscience suggests that this process rests upon (forward or “bottom-up”) prediction errors that ascend brain hierarchies from the low sensory levels to high levels of deep generative models (Carhart-Harris and Friston, 2010). For example, the number of “top-down” efferent neurons targeting the eye far exceeds the “bottom-up” afferent number ascending brain-ward. Descending predictions try to resolve prediction errors at each hierarchical level, thereby providing an accurate account of sensations, in a minimally complex fashion.

The FEP provides a model to think about belief updating and what this might entail. The binding of free energy equates to the resolution of prediction errors (i.e., surprise and uncertainty). Thus, the conversion of free into bound energy results from belief-updating to increase the accuracy–or decrease the complexity–associated with our beliefs about the world’s states of affairs.

In sum, Friston maintains that the brain’s main aim is to minimize “surprise”–as best it can.

Prediction error is minimized in two main ways:

1. *Action*, which reduces prediction errors by selectively sampling sensations that are the least surprising,^6^ thereby helping to approximate the organism to its environmental niche, or affordance (see below).
2. *Perception*. Changed perceptions follow from belief updating resulting in more reality-consonant *predictions*.

Both action and perception operate semi-instantaneously–in the twinkling of an eye. In the longer term, the structure of generative models are, in health, continuously being updated, especially their *complexi*ty. How this plays out in psychopathology are main themes of this article. Much of our focus will be on what Friston and collaborators call “structure learning” (Tervo et al., 2016; Friston et al., 2017; Gershman, 2017; Isomura and Friston, 2018), namely, learning the repertoire or narratives that constitute our prior beliefs–or hypotheses–about how our world works, and how these might be influenced therapeutically. Although the FEP applies to these structural priors, getting them right can be a tricky business. If we have too many prior hypotheses, our models are too complex and will not generalize in a capricious and changing world. Conversely, if we have overly simplistic models, with an insufficient number of priors to call upon we will fail to predict our sensations accurately. In both cases, free energy increases and we fail as self-evidencing creatures. We shall argue that psychopathology largely resides in the discrepancy between the experience of uncertainty and the paucity or defectiveness of procedures needed to reduce it.

It is important to note that minimizing surprise does not equate to stasis or clinging to the *status quo*. First, the internal milieu, i.e., physiology is constantly changing and so interoceptive prediction error will drive appetitive, safety-seeking, and reproductive behaviors (Seth, 2015) exploiting an innate system whose prediction postulates that “engagement with a source of uncertainty provides maximal opportunities to resolve that uncertainty” (Solms, 2019). Second, organisms live in constantly changing environments, both in the short- and long term, and need creative solutions to adjust and adapt to these. Integral to this is the invisible and imperceptible flux of time. This aspect of active inference can be thought of in terms of “time out” *simulations*. By uncoupling prediction and action, the mind models the possible outcomes of action in terms of expected surprise or uncertainty. Thus active inference furnishes building blocks for allostatic adjustment, i.e., “flexible information manipulation without the need to commit to particular decisions at an early stage of processing” (Knill and Pouget, 2004). Seen this way, imaginative exploration and innovation are no less surprise-minimizing than ingrained, self-perpetuating, ways of explaining the lived world. It is this former aspect that is built on and prosthetically enhanced in the social practices of psychotherapy.

## Psychoanalytic Resonances

At first encounter, this abbreviated account may seem to come from a conceptual universe far removed from psychoanalysis. Knowing our left from our right hand,^7^ active inference can no doubt reliably discount the chances of a west-rising dawn. But knowing about the physics of the world “out there” is surely a very different matter to the task of understanding oneself and other people? The argument of this paper is, to the contrary, that Fristonian principles apply equally, if not more so, to the interpersonal realm.

Consider a baby crying for its mother. At times, she is there on demand; at others, she is inexplicably delayed. In order to make good predictions, a theory of mind is needed–“maybe she’s tired, angry about my neediness, intoxicated, making a new potential rival with Dad.”

The Bayesian brain gradually–and with help–learns to infer the causes, affects, motivations, and meanings which shape the interpersonal world. Prediction error is built into this calculus; this will steer *actions*, aiming to minimize expected error and therefore, *via belief updating*, increase the chances of our predictions being adaptively correct:

“Mummy, I called you last night when I had a tummy ache, but you didn’t come! I thought you had gone away”

“So sorry darling, how horrid! I must have been fast asleep. If it happens again you must come through and wake me up.” (c.f., Allen et al., 2018)^8^

Here, the child is being taught the role of action (“come through”), interoceptive affect regulation (“So sorry–how horrid”), and a relevant hypothesis or prior (“maybe she’s asleep and can’t hear me”). Note the *conversational* or narrative aspect of prior/posterior interplay. *Vis-a-vis* the physical world, action is used to minimize the discrepancy between the organism’s model and environmental “affordances” (Dennett, 2017) that themselves can be purely epistemic–in the sense of resolving surprise and uncertainty. In the interpersonal world, dialogue is not so much with physical objects–moving one’s head to get a better view, etc.–but with the other, engaged in a reciprocal project of speech acts (c.f., Talia et al., 2014). At this level of the Bayesian hierarchy, prior beliefs are higher order cognitions (HOCs; Rudrauf, 2014; Debbané and Nolte, 2019), initially “borrowed” by infants from parents’ minds, based upon their caregiving disposition. We shall see how similar processes apply to psychoanalytic work.

This moves the Free Energy approach toward developmental and interpersonal conceptions with which psychoanalysis can begin to engage. Consider three relevant aspects. First, when it comes to precedence in the concept of free energy, Freud trumps Friston (Cahart-Harris and Friston, 2010; Solms, 2013). In the unpublished “Project” Freud (1895/1950) proposed the concepts of “Bindung” and “Entbindung,” i.e., energy “bound” and unbound.”^9^ Freud abandoned his “project,” as he moved toward more psychological models of the mind. However, in his 1911 paper *Formulations on the Two principles of Mental Functioning*, he differentiates primary process thinking, in which libido seeks discharge, from secondary processes which encompass language, sublimation, and ego-mediated restraint. The primary processes can be thought of as bottom-up impulses (interoceptions) stimulating and interacting with the top-down secondary process of affective modulation, verbal representation, and logic. For Freud the aim is homeostasis or psychic equilibrium, through binding, or if that fails, “discharge” in form of symptoms:

“The purpose of the mental apparatus [is] to keep as low as possible the total amount of the excitations to which it is subject” (Freud, 1925).

Relevant to our later discussion of trauma is emphasis on painful memories, which, if unregulated, remain disruptively unbound (Freud, 1895/1950). On the free energy view, this corresponds to unresolved surprise, uncertainty, or prediction errors–all which may be experienced as mental pain, and therefore part of the terrain of psychoanalytic therapy.

Another close parallel is between Friston’s model and Bion’s (1962) quasi-mathematical picture of how alpha function (i.e., maternal reverie generating top-down predictions) processes infants’ “beta elements” (uncontained, unnamed bottom-up raw experience) (c.f., Mellor, 2018). This “borrowed brain” (Holmes and Slade, 2017) model introduces a vital interpersonal dimension to the Bayesian process. Parental mentalizing–seeing, understanding, and resonating with their infants’ affects–is initially non-verbal and implicit: communicated by facial expression, tone of voice, affiliative touch, swinging rhythms of soothing, or stimulation. These embodied gestures present a model of the infant from the caregiver’s perspective, helping the child to integrate primary sensory signals (Fotopoulou and Tsakiris, 2017) into regularities of emotional and interpersonal consequences. In the context of increasing predictability, the infant explores the environment (beginning with the mother’s breast) and the mind of others with unconscious phantasies and proto-representations (i.e., building a repertoire of Bayesian “priors”). With the help of predictable input from the caregiver, the infant brain begins to differentiate self versus non-self causes of sensations that underwrite a sense of agency and the emergence of selfhood (Fonagy et al., 2002).

This leads us to a third Friston-Freud link: the analysis of *boundaries*. Bion postulated a “contact barrier” between conscious and unconscious thought, ensuring that phantasy is sharply differentiated from reality–the pleasure from the reality principles, the gratifying from the missing–and much-missed–breast.^10^

Comparably, from a FEP perspective, living entities possess a statistically permeable boundary across which occur exchanges–material and informational–with their surroundings. The mind is *bounded*; at one level, the “world” can only be known *via* its impression on the sensory epithelium and the belief updating entailed by active inference that sensations evoke. This boundary (known as a Markov blanket, see Kirchhoff, 2017; Kirchhoff et al., 2018) demarcates any system or creature from the environment in which it is immersed and also describes nested layers of top-down/bottom-up interfaces within the brain.

The “world” is opaque to the brain except insofar as it samples sensations from outside across the Markov blanket, matching them with its own internally generated models, identifying discrepancies as prediction errors and acting and/or thinking to minimize them. As seen (felt, smelled, heard, propriocepted) through a Markov blanket, “the world” is inferred, based on sensation: seeing, feeling, etc. is believing. Markov blankets are “nested,” in the sense that boundaries exist not just between the mind and its environment, but within the body-mind at different levels of complexity and immediacy. Bottom-up and top-down processes interact in a hierarchical Helmholtzean fashion throughout the nervous system. Thus, believing is also seeing.

Another connection between FEP and the preoccupations of psychotherapy is the role of the self. From a FEP perspective, the “inner world”–bounded and entropy-defying –necessarily entails a model of the environment (Conant and Ashby, 1970)^11^ and the organism’s place within it. This presupposes a rudimentary “self” however primitive or unconscious that representation might be.^12^ Enhancing the sense of self–active, authentic, aware, and apposite–is a key aim of psychotherapy.

## Bayes in Action

Let’s now look now at a quotidian example illustrating the Bayesian brain in action, and its relevance to psychotherapy.

One spring morning, in the course of JH’s daily run across agricultural land, he noticed that the farmer had recently sprayed weed-killer. As he ran, he experienced an unpleasant sickly smell and slight feeling of nausea. Worried that he might be adversely affected, as he had been in previous years, he returned *via* a detour. The following day, following the same course, the smell had gone, but he noted in his *peripheral vision* a dark flapping object. His first thought was that this was a bird, perhaps a crow, affected by the previous day’s poison; he turned his head to engage foveal/*central vision*, then *approached* to investigate further and if necessary rescue the creature. The closer he got to the “object” however, the more the putative stricken bird revealed itself to be no more than a fragment of wind-blown black plastic, a remnant of a discarded fertilizer bag.

This trivial incident illustrates a number of the Bayesian FEPs.

- JH’s slight feeling of nausea on the previous day, and knowledge of the hazards of weed-spraying, raised the “prior” probability of a “sick bird.” This “somatising” mind-set was based on the previous day’s nausea.
- The “*prior*,” or meaning attributed to this experience, based on *selective sampling* in *peripheral vision* and therefore error-prone, was guided by interoception (the feeling of sickness) and the epistemic affordance^13^ of looking more closely at the cause of sensations.
- The stimulus was ambiguous and, thanks to the inherent imprecision of peripheral vision, “noisy”; thus *free energy minimization* was required, *via*
  1. *Action*–turning the head and *moving toward* the “flapping” in order to disambiguate (c.f., Seth, 2015) and increase perceptual accuracy–reducing uncertainty and subsequent surprise.
  2. Belief updating–or hypothesis-revision (“the poison will have dispelled by today so it would be odd/anomalous if this really was stricken bird”).
- This *active inference*, led to a
- *Posterior belief*: a free energy-minimized explanation of reality, external (“it’s only flapping plastic”) and internal (“no more nausea; I’m not going to get ill”).

We shall return to this example in our discussion of transference.

## Mentalising

As already mentioned, integral to active inference is an organism’s “*sense of self.*” In humans and other primates, this implies the emergent property of self-awareness (Seth, 2015; Seth and Friston, 2016; Friston, 2018). The better we know who “we” are, the less likely we are to be entrapped in prediction error. Being able to model the consequences of our actions means, we have models of a counterfactual future, and thus to choose how we perceive the world and how to act on its affordances. The healthy brain is both prediction and action generator, constantly attempting to align perceived reality with internalized models (Bolis and Schilbach, 2017), including factoring in the self as a source of potential error and uncertainty. To the extent that psychotherapy helps its subjects to know themselves better, the more these processes will be enhanced.

FEP holds that nested Markov blankets operate “all the way up” (Kirchhoff et al., 2018). Thus, the search for self-awareness points to a further level of the top-down/bottom-up hierarchy (Wilson, 2002): *meta-cognition*, the capacity to think about thinking, or *mentalise* (Frith, 2012). Mentalising is the capacity to stand outside oneself and scrutinize one’s–and others’–active inference. The processes by which we populate our *umwelt* with objects, motivations and meanings operate below consciousness most of the time–until problems arise, as they inevitably do; given the complexity of the social and physical environments in which humans find themselves. This is especially true of the inherently unreliable nature of self-appraisal, and the related need to navigate the shared affective world of intimate others (see Rudrauf and Debbané, 2018 for the Projective Consciousness Model of such inference processes).

Frith (2012) argues that such metacognition is especially relevant to the cooperative or “we-mode” procedures, which occupy a great deal of human waking life. He cites a range of experimental evidence showing how inaccurate unmodulated self-appraisal can be–we cannot easily see ourselves as others see us. He has shown experimentally how two heads are better than one: “through discussions of our perceptual experiences with others, we can detect sensory signals more accurately.” (Frith, 2012)

Active inference, if carried out jointly, surpasses lone attempts to reduce prediction error and forestall entropic surprise. Developmental studies show how an “intimate other”–typically an attachment figure – knows our self better than we can we know ourselves, and it is through this joint appraisal that our internal self-model becomes progressively refined in the course of psychological development (Moutoussis et al., 2014; Palmer et al., 2015; Hamilton and Lind, 2016; Fotopoulou and Tsakiris, 2017). One of the roles of psychotherapy is to reactivate this process.

### “Duets for One”^14^

This dyadic self slant takes us to the question: what happens when two Bayesian brains interact? Friston and Frith (2015) stake out the maths of this, using birdsong as a paradigm for dialogic “conversations.” The authors base their discussion on the phenomenon of “sensory attenuation” (Brown et al., 2013), in which sensory feed-forward is inhibited during action, in order to preclude the log-jam that arises if bottom-up were to meet top-down *in medias res*.

This sensory attenuation is integral to “turn taking,” as a fundamental feature of human interactions, whether verbal or non-verbal (Holler et al., 2015). One can either listen or talk, but not both. In intimate conversations one can, through the other’s ears, “hear,” and so come to know oneself better. If each agent assumes the other is “like” themselves, the boundaries between them are temporarily dissolved. Listening, the sensory input of A (i.e., “language,” verbal and non-verbal) can be taken and “priored” (i.e., subjected to top-down predictions) as though it arose in B herself. This in turn leads to “action” (i.e., more speech), revised posteriors, and so on–a similar process applying to B *vis-a-vis* A. As Friston and Frith (2015, p. 14) put it, the result is

“a collective narrative that is shared among communicating agents (including oneself). For example, when in conversation or singing a duet, our beliefs about the (proprioceptive and auditory) sensations we experience are based upon expectations about the song. These beliefs transcend agency in the sense that the song (e.g., hymn) does not belong to you or me”

The resulting boundary dissolving synchrony of Friston and Frith’s birdsong model (i.e., “epistemic match”^15^) points the way to the nature of therapeutic conversations in psychotherapy. The therapeutic “duet for one” helps bind potentially disruptive free energy in creative ways, fostering psychological resilience. It also provides a neuroscience account of the psychoanalytic notion of the “third” (Ogden, 1994), the phantasy-imbued conversation which arises between two intimate participants (i.e., analyst and analysand), contributed to by both, but pertaining to neither.

## Free Energy, Attachment, and Psychopathology

Free energy minimization describes how organisms adapt to unpredictable environments, forming a bulwark against entropy, and a springboard for survival and flourishing. But the negentropy which characterizes living organisms is inherently fragile. Given an entropic world, as the Red Queen famously puts it, “*it takes all the running you can do to stay in the same place. If you want to get somewhere you must run twice as fast….*” (Carroll, 1871/2009).^16^

This fragility, arguably, is the basis of psychological illness/psychopathology. Things can–and do–go wrong in a number of different ways (Solms, 2015; Powers et al., 2018). First, there is the ever-present danger of “trauma.” Despite best laid plans, unpredictable, unforeseen, and deleterious environmental impingements can overwhelm prediction error minimization. As Freud put it:

“we describe as ‘traumatic’ any excitations from outside…powerful enough to break through the protective shield…and result in permanent disturbances of the manner in which the energy operates.” (Freud, 1925, p. 3)

The Markov boundaries (“blankets”) of body and mind form the basis for adaptive living. The environment is “taken in” in order that it may be appraised and evaluated but also kept at bay so that it can be manipulated to the organism’s advantage. The same goes for internally generated impingements, phantasies, demands, urges, or drives. In trauma, entropy, i.e., free energy unbound, takes over at a specific level of nested Markov blanket (for instance, the expectation of a safe or relatively predictable world). The mind is colonized by chaos and the potential for psychotic functioning increases if the thinking apparatus itself is overwhelmed, or as it might be put psychoanalytically, “attacked.” Trauma, from this perspective, exerts pressure for parameter adjustments in generative models to deal with increased complexity that arises from traumatic experiences (Hopkins, 2016).

Second, the capacity for active inference may be impaired. Active inference, as the term implies, depends on agency and belief-updating. Both are skills, acquired and honed in the course of development and reflecting the role of caregivers, and thus vulnerable to environmental disruption. It is this acquisition that underlies structure learning and building–in a familial and an encultured setting–the right sort of priors for explaining dyadic interactions with others and our own bodies.

Seen this way, psychopathology results either from the impact of overwhelming trauma, or when the capacity for active Bayesian inference is compromised. Here, the attachment ontogenetic schema for categorizing intimate relationships provides an evidential heuristic. Insecure attachments compromise active inference (Holmes and Slade, 2017): in the absence of an internal secure base (Holmes, 2010), exploration, physical and psychological, is curtailed. This limits the extent and range of sensory sampling of the environment, and so the variety of priors or hypotheses available to account for them. Both the “breaking” (i.e., creative destruction) of priors and the “making” (i.e., creative construction) of new ones are inhibited (c.f., Holmes, 2010; Leonidaki et al., 2018).

In anxious or “hyperactivating” attachment, agency tends to be absent or eroded. Rather than actively searching or changing their environment, sufferers remain passive in the face of loss, conflict, or trauma (Knox, 2010), a state famously described as “learned helplessness” (Maier and Seligman, 2016). Here, the self is suffused with unmodulated affect. In terms of structure learning, commitment to the single prior “nothing I do will change anything” precludes epistemic affordance and the testing of alternative hypotheses. By contrast, the hallmark of deactivating, or dismissive attachments is repression and affect suppression. While this yields a measure of niche-specific security, it also renders the individual vulnerable to unexpected trauma or interpersonal friction, as well as precipitating health-diminishing physiological changes.^17^

One of the “functions” of negative affect–fear, sadness, mental pain–is as *signals* of prediction error (c.f., Barrett, 2017; Solms, 2019), i.e., a discrepancy between top-down expectation and bottom-up signal–the wanted breast and the reality of its non-appearance. If, as in anxious attachment, negative effects are felt to be un-minimizable this may lead to–or indeed constitute–psychological illness. In deactivating attachments there is a trade-off between free energy minimizing and complexity reduction. By placing interceptions beyond conscious awareness–and so beyond mentalising–the learning of adaptive structural “priors” is precluded.

Disorganized attachment is a proven precursor to later psychopathology including Borderline Personality Disorder (Bateman and Fonagy, 2012). Two main reasons have been identified. First is the low threshold for interpersonal distress typical of such individuals, which means that mentalising and so top-down modulation–free energy minimizing–of negative affect are inhibited (Nolte et al., 2013). Second, sufferers typically experience from “epistemic mistrust” (Fonagy and Allison, 2014), resulting in difficulties with the collaborative mentalising/social learning “duets” described above (Nolte et al., 2019). In such a solipsistic world, deliberate self-harm, substance abuse or risky sex are self-soothing last resorts; however self-defeating. Bion’s (1962) “minus K”–i.e., the “active,” dynamically motivated wish *not* to know is also relevant. Selective sensory sampling (including interceptive input) which excludes new information means that simplistic, albeit dysfunctional models, of the world are maintained.

In all three patterns of insecure attachment, freedom is sacrificed for the sake of a degree of security. Freud defined neurosis as a turning away from reality. From a FE perspective, this can be seen in terms of attempts to bind free energy by reducing complexity. Fixed beliefs about the world are clung to, rather than updated in the light of experience. The more precision–which may be spurious–is afforded prior beliefs,^18^ the less likely are new experiences sought in order to update generative models. A degree of negative capability,^19^ or creative not-knowing–and hence the need for exploration and innovation–is thus built into the free energy formulation. In the Kleinian dichotomy, PSP (paranoid-schizoid position; Klein, 1946) represents a simplistic either/or good/bad model, while DP (depressive position; Klein, 1997) a more complex, whole and nuanced approximation to the world’s (epistemic and affective) affordances.

Parsimony^20^ plays an important role here, i.e., the need to reduce, Goldilocks fashion (neither too many nor too few), the chaotic multiplicity of possible predictions to a number of stable “attractors.”^21^ Such parsimonious models of the world must have value^22^, i.e., be of interest to the organism, and help with its project of survival, maintaining homeostasis, facilitating consciousness, staying safe, enhancing foraging potential, reproduction, etc. Their function ultimately is to minimize the affective manifestations of chronic prediction error.

On this reading, the free energy formulation is inherently motivational. This has psychotherapeutic relevance given that therapy is ultimately concerned with people’s needs, wishes, and wants (c.f., Hopkins, 2016). Moving toward complexity-reducing, parsimonious attractors that enhance interpersonal satisfaction–and eluding self-fulfilling priors (e.g., learned helplessness) are markers for psychological health.^23^ Our contention is that the procedures of psychotherapy, and especially psychoanalytic variants, are well placed to enhance these processes.

## How Psychotherapy Fosters Active Inference

### Bio-Behavioral Synchrony Reduces “Surprise”

Bio-behavioral synchrony (Feldman, 2015a) refers to the physiological, endocrinological, and behavioral entraining characteristic of care-givers and their infants, and their developmental sequelae–and can be seen as a prototype for life-long “duets for one.” The greater the synchrony in the first year of life, the more pro-social, exploratory, and less anxious the child is likely to be at school entry (Feldman, 2015b).

Bio-behavioral synchrony takes place during “sensitive periods” (Tottenham, 2014) in which immature individuals are open to affect co-regulation, with the help of their care-givers. Thus, in the classic “visual cliff” paradigm (Gibson and Walk, 1960), 1-year-old children are more adventurous and take greater risks if their mothers are seen to be encouraging and reassuring. This relational regulation is not confined to human mammals (Hofer, 2002). In the presence of their mothers, rat pups show interest in–rather than aversion to–strong odors, compared to those separated from their mothers at birth, and when mature show diminished startle reflexes and greater exploratory drive.

Secure attachment transmits epistemic trust as a springboard for social and physical exploration cross the life cycle. Coan et al. (2006) and Coan (2016) studied happily married couples in their “hand-holding” experiments. The wives were exposed to stress–the threat of a mild electric shock–while being observed in an fMRI scanner. Markers of HPA axis arousal were minimal or non-existent when holding their husbands’ hands as compared with facing the threat on their own. From a free energy perspective, prediction error is lessened in these dyadic scenarios. Instead of a fast track (Kahneman, 2011), low-precision “danger” attractor, in the “duet for one” scenario, the potential free energy of threat is minimized. The “victim’s” threat-induced arousal does not directly impact the hand-holding husband’s HPA axis, who is thereby able to bring “top-down” reassurance into the shared experience. Undertaken together, the whole mini-trauma becomes negligible. The husband’s bound energy pathways transmit the thought to his wife: “the experimenter is not really going to do anything nasty to us.”^24^

Clients entering psychotherapy have typically had reduced sensitive periods of affiliative learning in their developmental histories, or, worse, attachment bonds reinforced not by collaboration and pleasure but by aversive stimuli. (Hofer, 2002). Many, especially those with a history of disorganized attachment, are on “hair trigger” for overwhelming anxiety (Allen et al., 2008). They are in the grip of perceptual distortion and ingrained prediction errors, driven by the need for a modicum of attachment security, however dysfunctional. An early task therefore in psychotherapy is to re-establish a degree of bio-behavioral synchrony. The patterns and rhythms of therapy help with this, as do the joint attention and affective mirroring (Holmes and Slade, 2017) typical of secure attachments. The more disturbed the individual, the longer this is likely to take–and it remains a fluctuating process varying from session to session and moment-to-moment within sessions.

Action is the prime means for improving the prediction and predictability of sensory sampling and thus minimizing prediction error. Clients suffering from depression are often in the thrall of cognitive errors that dominate their affective world: “everyone hates me,” “I am useless,” etc. These self-perpetuating–albeit parsimonious–priors not only bind free energy but also undermine agency and the ensuing accuracy of predictions. Passive helplessness pervades, interspersed with depressive auto-denigration. The “hand-holding” help of a therapist fosters action, initially in the form of verbal exploration. When things go well, depressive priors begin to be revised in the light of experience.

Bio-behavioral synchrony and the fostering of agency are probably common to all effective therapies. The remainder of our discussion focuses a free energy perspective on psychoanalytic therapies. Here, the role of “action” is less evident compared, say, with cognitive behavioral therapy (CBT), although the impulse to act–or “act out”–is an important focus for transferential and counter-transferential work. Indeed, choosing to seek help for psychological difficulties in itself implies a degree of agency. Furthermore, if conversation is seen in terms of “speech acts” analytic dialogue is in itself agency-enhancing.

## Decoupling

We will touch on a number of key features of the analytic approach: free association, dreams, sexuality, reflective discourse, transference, and mentalising. All depend on “*decoupling*”–introducing a degree of “play” into the bottom-up/top-down surprise-minimizing articulations of everyday life (c.f., Holmes and Slade, 2017). In the presence of a modulating, moderating, affect-buffering therapist, surprise/energy unbound becomes tolerable and, when therapeutically scrutinized, extends the repertoire and range of a person’s counterfactual realities, i.e., priors. Built into this model is both “creativity” and “destruction,” in the sense that modification of error-prone priors entails their replacement with alternative hypotheses. The greater the range of prior hypotheses, the greater the opportunities for error-minimized binding and the less the need to resort to rigid, limited, or anachronistic priors, at the different levels of a hierarchy of generative models. This, in turn, enhances the adaptedness of the sufferer to their environment, including, *via* mentalising, the self. Part of the process makes the patient’s model more accurate by revised belief-formation, and part by complexity reduction, especially in relation to resolution of conflict and trauma (Hopkins, 2016).

### Decoupling From “Below”: Free Association

Reducing prediction error is a complex multi-level and recursive process that reverberates up and down a series of interconnected message-passing hierarchies. “Bottom-up” does not refer simply to activity at sensory epithelia, but at each level of synaptic connection in a nested hierarchy of message-passing within canonical microcircuits throughout the brain. For example, Lanius et al. (2015) discuss decoupling between Prefrontal Cortex (PFC) and amygdala in post-traumatic states, and how, in the absence of top-down input from the PFC, patients attempt to dampen amygdala activity by resorting to substance abuse or self-harm. Observing these processes in a therapeutic setting forms a first step toward establishing reconnection and enhancing modulation of raw affect.

Barratt (2016) argues that Freud’s greatest discovery, clinically and theoretically, was the concept and practice of “free association.” Freud’s (1916) image of this was that of the passenger in a train looking out of a window and observing the view as it flashes past. In free association, thoughts, interoceptive bodily sensations and effects, impulses, and images enter the mind “from below.” As analysand and therapist collaboratively enter states of free-floating attention and negative capability, top-down constructions are temporarily set aside. Avoidant clients, with intellectual defenses, are both resistant to, and especially likely to benefit from joint attention to such free-associative experiences. With their co-regulatory sensitive period re-opened, they can explicitly attend to repressed feelings and fears. Free energy can now be minimized through prior modification and simulated action rather than repression. As in the study by Coan et al. (2006), the therapist’s calming, containing, “slow-thinking” conversational presence generates “forms of feeling” (Mears, 2018), which the sufferer can discern and grasp rather than fearfully evade.

### “Action Replay”

A crucial technique in the mentalising approach to psychotherapy with people suffering from Borderline Personality Disorder is a procedure known as “pressing the pause button” (Allen et al., 2008), when therapist and client interrupt the flow of their interactions in order to examine “what was going on between us just now.” This disrupts automatic top-down/bottom-up pathways, making thoughts and behaviors available for scrutiny. An “event”–e.g., a client’s sudden outburst of anger triggered by a therapist’s holiday–may stimulate prolonged collaborative reflection, encompassing previous comparable interpersonal experiences. The client begins to tease out differences between a therapeutic “break” with a high probability of resumption, and a childhood history of being arbitrarily abandoned, leading to more complex and realistic posteriors about the reversibility of loss.

### Dreams

During hours of dark, prediction errors inevitably increase. Applying the free energy model to the neurobiology of sleep, Hobson and Friston (2012) suggest that, when dreaming, bottom-up sensory input and top-down prediction are de-coupled. In the absence of afferent input, potentially free-energetic–and so entropic–memory traces of the “days residue”^25^ can be “bound” into parsimonious representations. *Via* synaptic pruning and consolidation of themes of affective significance, this “housekeeping” process reduces the chaotic complexity of everyday waking life.

Although this approach does not fully endorse the Freudian notion of dreams as disguised wish fulfillments, it sees dream themes as value-laden, replete with affective saliences which have not reached waking conscious awareness (c.f., Solms, 2013). At the same time, dreaming embodies *counter-factual simulation* or *virtual reality generation*. Triggered by the day’s residue, possible future scenarios are played out in dreams helping to build a repertoire of free-energy minimizing priors, able to reduce prediction error when encountering future potentially traumatic events.^26^ This process does not forestall emotional pain, but safeguards against, or at least postpones entropic surprise. Anything and everything is possible, thereby arming the dreamer against the unpredictability–improbabilities–of life.^27^ Freud excluded undisguised trauma-related dreams from his wish-fulfillment theory. From a free energy perspective, dreaming reworks trauma so that it becomes “thinkable”: “only a dream,” or “that was then, inescapable, horrible; this is now, still painful, but tolerable” (Kinley and Reyno, 2017).

### Transference

In the “flapping black object” example, an ambiguous stimulus presented itself to the subject, who saw something “*untoward*” out of the corner of his eye. Given the high degree of imprecision intrinsic to peripheral vision, this was interpreted in the light of plausible “prior” based on the previous day’s experience–a possible poisoned bird. Disambiguation (Seth, 2015) followed: face-forward movement *toward* the object led to a revised “posterior.” This illustrated how an interoceptive anxiety (“my nausea suggests that the bird could also have been poisoned by the weed spray”) could shape an erroneous prior, leading to a maladaptive “perception,” in which a picture of the world appropriate to the past (here the previous day) was carried over, or *transferred*, inappropriately, into the present.

According to Laplanche (2009, p. 93) “the analyst is the one who guards the enigma and provokes the transference.” In his terms, the analyst is–like the world glimpsed in peripheral vision–an “enigmatic signifier,” not perhaps an entirely “blank slate,” but nevertheless embodying the reticence –creative ambiguity–inherent in analytic technique. Drawing on that ambiguity for therapeutic ends, the analyst receives and helps identify the patient’s projected object relations or unconscious phantasies.^28^

From a free energy perspective, transference is an entrenched “prior,” inaccessible to updating *via* active inference. In the classic Kleinian concept of “projective identification” (PI) (e.g., Ogden, 1992/2018), transference is jointly *enacted* by therapist and client. PI can be conceptualized in free energy terms as an attempt to *shape* the interpersonal world in the light of pre-existing phantasies, rather than to revise priors in the light of experience. For example, a therapist might “forget” to inform a PI-driven client about an upcoming break, having been induced unconsciously by the client’s expectation of abandonment actually to do so.

But–exemplifying psychoanalysis’ paradoxical capacity to snatch success from the jaws of defeat–such enactments also have the potential, as Winnicott (1974, p. 107) puts it, to “bring trauma within the arena of omnipotence” and hence be available for therapeutic work. The FEP point here is that one way to minimize surprise is actively to shape or seek out environments in accordance with one’s priors, thereby eliminating the necessity to update them. Recognizing and exploring projective identification observes this process in action and offers a more flexible range of options for living out one’s relationships. A crucial prerequisite is the therapist’s countertransference awareness (Brenman Pick, 1985, 2018)–the capacity to be objective about one’s own subjectivity.

### Sexuality

The FEP is inherently temporal: *sensation* stimulates a prior, leading to *perception* and, *via* active inference, posterior *revision*. In the example, it was a “relief” to realize that the putative bird was a figment of imagination. In this FEP account, there is an affective arc of motivated tension, consummation, and resolution, in which the very binding of energy is rewarding. By deepening trust and discouraging premature closure of surprise, therapy fosters this expansion of the realm of desire.

Put another way–ambiguity and its resolution is both *exciting* and *rewarding*. To return to Laplanche (1987), enigma–which can be reformulated in this context as prediction error–is central to this process. In his neo-Oedipal model, the “breast” is a “sexual organ,” but, for the naïve infant, one wrapped in mystery. The mother’s loving sensuality in relation to her baby is suffused with a degree of eroticism which the child cannot fully comprehend.

Building on this, Target (2007) suggests that sexuality is the outstanding exception to the observation that joint attention and accurate affect-mirroring by caregivers underpins the development of the child’s sense of self (Fonagy et al., 2002). In the realm of genital sexuality, parents typically distract, avoid, or punish rather than directly reflect the child’s explorations and feelings of excitement. This, Target argues, leaves a residue of *mirroring-hunger*, whose resolution is postponed until sexual life begins in adolescence, and a suitable partner/other is found with whom a sexual duet for one can begin to be played. With its recurrent rhythms of desire and resolution, sexuality remains suffused with a continuing ambiance of enigma. Part of the mystery and paradox of sex is the tension between the fact that one can never fully “know” the other, and yet, through sex (genital and in any of the ontologically derived adult expressions of infantile, polymorphously perverse sexualities), one approaches their intimate being.^29^ In FEP terms, sex “plays” with energy bound and unbound and their relationship to, among others, the reward system.

When sexuality permeates the analytic relationship as erotic transference, the “decoupling” virtual reality ambiance of psychoanalytic work, enables such feelings to be jointly mentalised, thereby enabling clients to develop a more explicit sense of the lineaments of their desires.

### Therapeutic Conversations

While underpinned by pre-verbal bio-behavioral synchrony, psychotherapy is in essence a specialized form of conversation, or proto-conversation (Mears, 2018). Based on Strachey’s (1934) classical paper on the “mutative interpretation,” Lear (2011) suggests that change in psychoanalysis relies on the interplay between two conservational vectors. First is the mirroring and role-responsiveness as the analyst enters into patients’ “idiolect,” helping to delineate their unique way of seeing world and self-stamped vernacular, always trying to find the right words to capture the patient’s “forms of feeling,” without imposing her or his own emotional vocabulary. At this stage, from the patient’s point of view, the top-down/bottom-up process runs smoothly, and, from a free energy perspective, un-“surprisingly.”

But at some point, a discrepancy (or ambiguity) will inevitably arise, as the analyst fails to conform to the patient’s top-down expectations. In the Strachey’s 1930s account, the feared punitive father turns out to be benign; in a contemporary version, a patient’s view of her analyst as abusive (“*you’re just getting off on my misery; you don’t really give a damn*”) might be confounded by a degree of compassionate and committed concern. Conversely, patients’ assumption that their therapists will be all-loving or all-forgiving comes up against confrontations, inflexible endings to sessions, the need to pay fees, etc. In the face of this discrepancy between desire and reality, patients do their best to maintain the *status quo*, clinging to past assumptions, attempting to evade the need to bind free energy with revised priors. This discrepancy then becomes the *point d’appui* of psychotherapeutic work.

From a free energy perspective, psychological ill health implies simplistic top-down models, and/or restricted sensory sampling, while structured complexity, as opposed to chaos or rigidity, is a mark of psychological health. Psychotherapy aims to increase the repertoire of its subjects’ models of themselves and their environment. It is no mean task for analysts to challenge their patients, to break the mold of maladaptive energy binding, and to move psychic structures toward this augmented complexity. It is tempting to collude, “supportively” maintaining the *status quo*, or gratefully (if silently) accepting the drop-out of a “difficult” patient. Yet from a Bayesian perspective, Moutoussis et al. (2018) suggest that complexity is crucial to treatment success: too much, and there is no generalization from good therapeutic experiences to blighted everyday lives; but if complexity is simplistically minimized, this inhibits the risk-taking and “negative capability” needed for psychic change.

Recent research by Talia and his group (Talia et al., 2014, 2018) lends further experimental support to this model and to the attachment categories discussed earlier. Analyzing transcripts of psychotherapy sessions, they show how the nature of therapeutic dialogue depends on the attachment status of both client and therapist. Securely attached clients–and therapists–engage in turn-taking “duets,” in which there is contact seeking, free exchange and modulation of affect and ideas. By contrast, insecurely attached people typically rebuff mutative speech acts. Their dialogue tends to be non-relational, with little affect-modulation, frequent backtracking, and repetitive interactive patterns.

The partial or occasionally total impasse created by these insecure speech patterns then becomes the focus of therapy. Painful affects–anxiety or misery–signal prediction errors, misalignment between wish and reality. But rather than leading to change, these become chronic and embedded. Psychotherapy mobilizes the active inference needed to resolve the impasse. The therapist enjoins the client to look at–mentalise–what is happening between them. Knowing that his or her hand is being metaphorically held, and that energy binding can be temporarily left to the therapist, the client can become more adventurous. In “duet for one” moments, initially fleetingly, therapist and client “sing” in ways that pertain to each and neither participant. Classical analytic geometry may encourage this–prone, in the absence of visual contact, patients take their analysts as part of themselves, drawing on the other’s “priors”–i.e., verbal “interpretations”–to widen the range of available top-down models of the world and its possibilities.

## Conclusion

In a perhaps slightly disingenuous moment of self-doubt, Friston (2010, p. 9) asks:

“What does the free-energy principle portend for the future? If its main contribution is to integrate established theories, then the answer is probably ‘not a lot’…[But it] could also provide new approaches to old problems that might call for a reappraisal of conventional notions.”

Wiese (2015) argues that while FEP may in a Popperian sense be “unfalsifiable,” it nevertheless represents a Kuhnian new paradigm. Our enthusiasm for the free energy model comes from the position of psychotherapy ecumenicalism (c.f., Holmes, 2002; Wampold, 2015; Holmes and Slade, 2017). We have argued that recovery from psychological ill-health is associated with enhancing the capacity to bind free energy and thereby facilitate prediction error minimization. Therapeutic procedures which foster these will be likely to be helpful, whatever their espoused brand name. These include the following: promoting agency; broadened sensory and interoceptive sampling, whether through CBT “experiments” or psychoanalytic free association; widening counter-factual simulation and the range of top-down hypotheses through dream-analysis and transference work; and fostering the capacity to modify priors in the light of experience, especially through the analysis of transference.

We have outlined some of the established interpersonal procedures which pave the way for these: bio-behavioral synchrony, epistemic trust, and turn-taking duet-for-one dialogue. From a research perspective, these features can be operationalized as benchmarks for assessing psychotherapy efficacy and procedural compliance. They help concentrate therapists and their supervisors’ minds, and, we predict, improve clinical outcomes.

A final point in favor of the FEP is that it conceives psychotherapy, not as an esoteric concoction, but as a “natural kind,” a specialized form of a general cultural phenomenon. Many aspects of cultural life–play, music, sport, drama, and iconography–depend on the top-down/bottom-up “decoupling” and mentalising which foster prediction error minimization,^30^ and so enhance recovery and resilience.

The homeostasis–psychological no less than physiological–essential, in Claude Bernard’s (1974) famous phrase, to a free life, is vulnerable to the ever-present forces of entropy. The discrepancies between the affordances of the environment–which in our species’ case is primarily interpersonal–and our inner models is the basis of prediction error, signaled by affective distress, leading, if unrevised, to entrenched mental pain or psychological illness. Learning to experience and resolve prediction error depends on the generative possibilities of intimate relationships. Where those fail or falter, psychotherapy provides a vital route to repair.

## Author Contributions

All authors listed have made a substantial, direct and intellectual contribution to the work, and approved it for publication.

### Conflict of Interest Statement

The authors declare that the research was conducted in the absence of any commercial or financial relationships that could be construed as a potential conflict of interest.

The handling editor declared a shared affiliation, though no other collaboration, with one of the authors TN.

## Footnotes

^1^We believe that this attempt to elucidate the “neuronal” basis of effective psychotherapy exemplifies the normal course of scientific progress. Darwin knew no more about DNA than did Freud about the fMRI-unveiled brain.

^2^Sterling (2012) has introduced the term “allostasis” to capture a more dynamic version of homeostasis in which an organism anticipates change in the internal milieu and sets about counteractive processes and actions.

^3^A concept that can be aligned with the psychoanalytic notion of “repetition compulsivity” (Barratt, 2016), or, more poetically, Wordsworth’s child as father of the man.

^4^The terms derive from Kant’s *a priori* and *a posteriori*.

^5^Surprise is defined as the *negative log-probability of an outcome*, i.e., how “likely” or “unlikely” a particular event, from a specific organism’s viewpoint to occur. The brain cannot compute “surprise” as such, but free energy *can* be evaluated and by “active inference.” Active inference depends on two key processes: modifying sensory input “bottom-up” from sensory epithelia, including the interoceptive, affect-triggering receptors, (Barrett, 2017), and “top-down” from the cortex – and at intermediate levels in between.

^6^This a key point of intersection between Bayesian predictive processing theories and “embodied enactive” models of the mind which prevail in cognitive science (Hohwy, 2013; Kirchhoff, 2017).

^7^Many metrics, affective and cognitive, start from the body orientation (Lakoff and Johnson, 2003).

^8^For a recent example of a simulated infant learning about mother’s quality of caregiving under active inference, see Cittern et al. (2018).

^9^Freud, well versed in classical literature, would have been familiar with Aeschylus’ play *Prometheus Bound*, and perhaps with Shelley’s subversive version of the myth, *Prometheus Unbound.*

^10^The latter two distinctions representing rudimentary generative models which, as unconscious phantasies, gradually become imbued with psychic meaning.

^11^See Seth (2015) for a discussion of the psychiatrist Ross Ashby’s early contributions to FEP.

^12^C.f., O’Keefe (1978) who discovered “place cells” in the hippocampus which, like an internal GPS, tells mammals where they are in their world. Knowing “who” we are entails, among other information, knowing “where” we are.

^13^Gibson defines affordances as “The *affordances* of the environment are what it *offers* the [individual], what it *provides* or *furnishes*, either for good or ill… [The word affordance] implies the complementarity of the [individual] and the environment.” (Gibson, 1986, p. 127). An “epistemic affordance” refers to the *meaning* of an object or event in the environment, in this case a “dark flapping object.”

^14^A phrase borrowed from Kempkinsy’s play of the same name and later film, a thinly described depiction of the life and illness of the cellist Jacqueline du Pre – including the questionable role of her psychiatrist!

^15^Fonagy and Allison (2014) argue that relaxing epistemic vigilance is achieved in normal development through “prefacing” one’s communicative intents with ostensive cues. This validates the recipient as a subjective, agentive self. Once epistemic trust is stimulated in this way, the channel for the transmission of knowledge – learning about minds – is opened and an *epistemic match* (Fonagy, personal communication 2018) can be created whereby one’s imagined self-narrative or feeling state can be recognized in the way the other communicates their version thereof.

^16^The “Red Queen hypothesis” in evolutionary biology (Ridley, 1993) is used to account for the apparently wasteful phenomenon of sexual (“twice”) as opposed to asexual reproduction.

^17^Avoidant infants separated from their care-giver appear unperturbed, but demonstrate raised cortisol and pulse rates suggestive of physiological stress (Bernard et al., 2013) with potentially long-term adverse health implications.

^18^Thus, OCD can be thought of in FE terms as fruitless striving for spurious certainty. In a riposte to Socrates’ much-quoted aphorism that “the unexamined life is not worth living,” Dennett reminds us that “the over-examined life is nothing much to write home about either” (Dennett, 2017, p. 278).

^19^Keats’ phrase to define the creative mind, popularised by Bion and much espoused by dynamic psychotherapists (e.g., Symington and Symington, 1996).

^20^Russell’s (2001) version of the Occam’s razor principle of parsimony is: “Whenever possible, substitute constructions out of known entities for inferences to unknown entities.” Thus, do we try to calibrate how new experience A is “like” known event B – and how it differs.

^21^In the mathematical analysis of non-linear systems, attractors are the set of numerical values toward which a system tends to evolve, from a wide variety of starting conditions. There is a possible link to the psychoanalytic notion of “fixation.”

^22^For a detailed account of system/ego-centric, subjective values, and their role in transitions from proto to truly mental states as well as precision-weighed uncertainty representation, see Solms (2019).

^23^C.f., Einstein: “everything should be made as simple as possible, but not simpler” (Reader’s Digest July 1977).

^24^The notorious “Milgram” (1974) experiments can be thought of in comparable terms. Those able to resist the seemingly sadistic urgings of the experimenter were using agency and top-down internal feedback–“I am under no obligation to continue with this.”

^25^Freud’s term.

^26^This account of dreaming can be compared to immunization in which overwhelming infection is prevented *via* prior exposure to attenuated forms of potential pathogens.

^27^Another parallel is with Bayesian weather forecasting. In the “numerical modeling method,” the computer, “top-down,” generates a large number of possible future weather patterns based on small differences in prior assumptions (Seth, 2014). Accuracy of priors is iteratively improved by posterior revisions which feed into the next day’s forecast, and so on.

^28^E.g., Patient: “*have* you *got any children*?”; Analyst: “*that’s a really interesting question–I wonder what has prompted it to come up today*?”.

^29^FEP accounts for the impossibility of self-tickling (e.g., Hohwy, 2016) on the grounds that top-down priors thwart the necessary unexpectedness of a tickle. A similar argument could be mounted to explain the unsatisfactoriness of masturbation as opposed to relational sex.

^30^The actor-audience divide decouples meaning from action in a variant of Coan’s hand-holding. Watching Shakespearean tragedy (Holmes, 2018)–or indeed a “horror movie”–extends the repertoire of top-down priors available for energy binding if and when real-life trauma strikes.
